# Insight into the main determinants of the struggle against overweight and obesity in Chile: Use of random forest techniques and econometric models

**DOI:** 10.1371/journal.pone.0309351

**Published:** 2024-12-16

**Authors:** Francisca Romo-Muñoz, Rodrigo Romo-Muñoz, Sebastián Niklitschek-Soto, Cristhian Aguilera-Carrasco, José M. Gil

**Affiliations:** 1 Magister in Statistics, Department of Statistics, Universidad de Concepción, Concepción, Chile; 2 Business Management Department, Universidad del Bío-Bío, Chillán, Chile; 3 Economics, Agri-Food Management and Population Health Research Group, Universidad del Bío-Bío, Chillán, Chile; 4 Department of Statistics, Universidad de Concepción, Concepción, Chile; 5 Department of Electrical and Electronic Engineering, Universidad del Bío-Bío, Concepción, Chile; 6 Center for Agro-food Economy and Development (CREDA-UPC-IRTA), Castelldefels, Barcelona, Spain; Universidad Diego Portales, CHILE

## Abstract

Overweight and obesity are considered the greatest public health problem in this emerging country, which worldwide has the second-highest percentage of overweight people among its population. The objective of this work was to analyse to what extent factors traditionally used in the study of overweight and obesity (such as sociodemographic and behavioural) and new variables proposed in the literature (such as stress, financial stress and emotional support) explain this disease in the adult population of Chile. Data were obtained from the III National Health Survey (ENS) administered by the Ministry of Health of Chile in 2017. The ENS collected a large amount of data with extensive geographic coverage. The survey comprised 4 questionnaires with a total of 576 questions, which were applied to a representative sample of the population in Chile. A double complementary methodological approach was adopted. A random forest (RF) classification model was used, and based on the results obtained, an econometric model of the censored dependent variable, specifically the Heckman sample selection model, was specified and estimated. The RF results allowed, for each of the factors considered in the research, the selection of variables with the greatest power to classify the individuals in the sample on the basis of nutritional state (normal weight, overweight or obese). Subsequently, the estimation of the parameters of the Heckman model made it possible to quantify the variables that most affected overweight and obesity. Most of the variables that make up the factors were found to be significant. Interestingly, psychosocial variables effectively influence overweight and obesity. In addition, the results for reviewing nutritional information and reviewing food warnings allow us to reflect on the impact that recent food policies have had on the Chilean population. The combination of RF and an econometric model allowed us to capitalize on the strength of both models to better explain the complex phenomenon of overweight and obesity. This approach allowed us to more accurately confirm the impact of traditional factors on overweight and obesity but to show also that other psychosocial factors are relevant and should be consider in future studies.

## 1 Introduction

In 2006, the World Health Organization (WHO) indicated that overweight and obesity reached global epidemic levels; more than 1 billion adults were overweight, and at least 300 million of them were obese [1]. Despite appeals and awareness campaigns, the prevalence of overweight and obesity has almost doubled (more than 1.9 billion adults aged 18 and over), among whom more than 650 million are obese, accounting for approximately 13% of the world’s adult population [2]. Struggles against overweight and obesity have become great challenges for the global health system [3].

The prevalence of overweight and obesity has become an important public health problem that generates high health expenditures; the literature has shown the close link between obesity and the probability of suffering from chronic noncommunicable diseases (NCDs), such as diabetes, some types of cancer and cardiovascular diseases [4]. The economic impact of overweight and obesity was estimated at 2.19% of world GDP [5], with the United States suffering the greatest impact associated with overweight and obesity, reaching 9.3% of GDP [6]. Additionally, overweight and obesity generate a decrease in productivity and indirect health costs, which have been estimated at up to US $1,000 in the United States for 2020 [7].

In Chile, according to data collected in the III National Health Survey of the Ministry of Health (MINSAL) of Chile [8], 74.2% of the population aged 15 or over is overweight or obese (39.8% and 34.4%, respectively). Among children under 15 years of age, the prevalence of overweight and obesity is 52% [9]. With these figures, Chile ranks as one of the leading Organization for Economic Co-operation and Development (OECD) countries in terms of the incidence of overweight and obesity, surpassed only by the United States [3]. This problem generates a significant economic burden for Chile, where 7% of health expenditure is used to combat overweight and obesity, which, on the other hand, has a negative impact in the Chilean GDP for about 3.8% [10].

Aware of this situation, the Ministry of Health of Chile has been working on the definition and implementation of policies to address this public health problem. These policies focus on promoting the consumption of healthy foods and encouraging physical activity. In 2000, the National Council for the Promotion of Health Vida Chile was created but was subsequently replaced by the Global Strategy against Obesity (EGO-Chile) in 2006, continuing until 2010. In 2008, the Healthy Eating and Physical Activity Program (PASAF, acronym in Spanish) for the Prevention of Chronic Diseases in Children, Adolescents and Adults was designed. In 2011, the Healthy Living Program was created and replaced the PASAF), and in 2013, Law 20 670 was approved, creating the Choose Healthy Living system, which is in effect to date. In parallel and as a complement to the previous legislation, in 2012, one of the strictest regulations in relation to food labelling was enacted ("Law 20 606 on Food Labelling"); the law was applied in 3 stages of gradual exigency in 2016, 2018 and 2019. Likewise, in 2014, an additional tax was established on sugary drinks (18%) and was included in the Tax Reform (Law 20 780). In 2016, the 2016–2025 National Policy on Physical Activity and Sports was established. Most recently, in 2018, the National Food and Nutrition Policy was created by MINSAL [11].

Despite the ongoing efforts by MINSAL, effectiveness has been questioned because the prevalence figures have continued to increase. However, it also unknown whether the figures would be higher if the above measures had not been adopted. In any case, the adoption of effective policies should be based not on criteria of political convenience but on a deep understanding of the issue and its causes as well as the main determinants of the prevalence of overweight and obesity in Chile.

From the literature review carried out, there is a consensus among researchers that the analysis of the main determinants of the prevalence of obesity is complex because it involves a web of factors. Traditionally, genetic factors [12, 13], behavioural factors [4, 14, 15], environmental factors [16] and sociodemographic variables [17, 18] have been considered. New research suggests that to better understand this phenomenon, it is necessary to incorporate complementary factors such as psychosocial factors, such as stress [18–20], financial stress or emotional support [21].

As can be observed, the phenomenon of overweight and obesity is complex to investigate, and traditional approaches such as parametric multiple regression models have some limitations, requiring more sophisticated analysis methods. Machine Learning techniques offer several advantages when conducting complex analyses with a large number of variables and observations. Random Forest (RF) is a non-parametric algorithm within Machine Learning that has demonstrated high performance in analyzing complex phenomena, although it also has some limitations. Numerous studies have shown that, when studying complex phenomena, the complementary use of Random Forest and econometric models creates a synergy that allows capturing the advantages of each method and overcoming their respective limitations [22].

The main objective of this work is to analyse the relative importance of different factors in the prevalence of overweight and obesity in Chile to provide relevant information that allows decision-makers to design the most effective measures. To achieve this objective, a novel methodological approach is proposed that combines a machine learning (ML) technique, i.e., random forest (RF), and stochastic econometric models, serving as the main contribution of this work. Likewise, it is one of the first studies that, based on the available information, considers a wide range of possible determinants of obesity and their possible interactions (most of the literature to date focuses on only a few factors related to genetics, metabolism and sociodemographic variables).

The rest of the paper is structured as follows. A review of the literature is presented below. In section 3, the data used are presented. In section 4, the methodological approach is addressed. In section 5, the main results are provided. The last section presents the conclusions of the study.

## 2 Literature review

Despite the diversity of studies available, there is a consensus among researchers that overweight and obesity are diseases generated by multidimensional causes such as those already mentioned in the previous section. Genetic factors, i.e., factors that come from parents’ genes, have been the least addressed in research worldwide and by public health policies in Chile because although there is indeed a genetic predisposition in weight gain [12], genetic factors have a limited impact in explaining overweight and obesity [13]. These factors also have limited impacts in Chile because historically, the main problem faced by the public health system was child malnutrition, decreasing from 37% to 11.5% between the 1980s and 1990s and then considered eradicated in 2000 with a prevalence of 2.9% [23].

Behavioural factors are associated with the adoption of healthy lifestyles (HLs) by the population, for example: a balanced diet (which is considered to be the easiest to adopt and the most studied in overweight and obesity); physical activity; and avoidance of tobacco and alcohol consumption (with the exception of the moderate consumption of red wine) [4]. In this study, the list of behavioural factor have been chose from to previous studies for the WHO [15], and Chile [14]. To determine the quality of the diet, the criteria established by the World Health Organization were used, which have been applied in other studies of healthy lifestyle habits in Chile [4]. This same approach has been applied in this research.

Environmental factors refer to the environments in which a person interacts. For children, these are home, school, and community settings [16]. In Chile, a list of the main elements of environmental factors has been defined, including the composition of the family group; the level of education of the parents; mother’s employment status; ways in which meal times are structured; the use of electronic devices; and the context where food is provided (restaurants, schools and home) [14].

Sociodemographic variables are particularly suitable for the study of overweight and obesity because social determinants model behaviour at the individual level and delimit the behaviours of people, even being collected for the context of overweight and obesity in Chile [17, 18]. For example, it is documented in the literature that the options of a healthy diet and opportunities for physical activity depend to a large extent on the economic resources that people have [18]. These elements can be classified as follows: sociodemographic characteristics (age, gender, race and ethnicity or nationality); socioeconomic status (education level, income level, type of employment, and household size); geographic sector of residence (urban, rural, communes, cities or regions); type of dwelling; employment situation; and pension system.

Research focused on overweight and obesity has been shown to be quite dynamic over time. New research suggests that to have a better understanding of overweight and obesity, it is necessary to incorporate new factors into the analysis. Psychosocial factors comprise stress, emotional support and depression [18]. Numerous studies have found a strong association between psychosocial factors and being overweight or obese [19, 20]. These psychosocial factors can occur in the family nucleus (divorce, bad marital relationship, poor mental health of the spouses, chronic diseases, domestic violence, child abuse, or tension in the family relationship) or they can be at the individual level (risky behaviour or poor mental health) [20]. In a complementary way, some recent investigations have suggested that financial stress is a determinant of overweight and obesity. Financial stress in a family occurs when there are high levels of expenses in relation to income; they have limited assets; and there is an inability to pay household bills or make necessary purchases [21]. Although this factor is closely related to the socioeconomic level of a person, different studies have found an association between financial stress and being overweight and obese [21]. Emotional support is also part of the determinants of overweight and obesity, and there is evidence that households lacking emotional support for younger children are associated with overweight and obesity [21]. Emotional support includes empathy, trust and help that one person offers to another in a self-less way so that they can develop self-confidence and have control of their own emotions. The lack of this help within the family nucleus is related to negative physical and mental health outcomes for all members of the household [21].

Recent literature indicates that when investigating problems characterized by complex relationships between variables and a multifactorial nature, the combined use of techniques such as Random Forest and econometric models yields superior results compared to using each model independently [22, 24]. The analysis of large datasets across various fields has been extensively researched, comparing different Machine Learning techniques and econometric models, which have steadily gained interest among researchers. Significant areas of scientific interest include theoretical analysis, finance, and stock markets. However, in the health sector, particularly concerning overweight and obesity, these combined approaches have not been widely utilized. More detailed accounts of research in these areas can be found in Pérez-Pons et al. and Shobana et al. [22, 24]

## 3 Materials and methods

To achieve the objective of this research, data from the III National Health Survey (ENS), prepared by the Department of Epidemiology of MINSAL and applied in 2017 were used [8]. The survey consisted of 576 questions distributed in 4 questionnaires. Questionnaire 1 comprised 21 modules corresponding to sociodemographic, behavioural, environmental and psychosocial questions and to specific questions about relevant health aspects. Questionnaire 2 comprised 13 modules corresponding to questions related to alcohol consumption, blood and urine sampling, and biophysiological measurements, among other topics. Questionnaire 3 addressed mental health, and responses to questionnaire 4, which addressed child development, were provide by the main caregiver in households with children between 7 and 59 months of age. Details regarding the questionnaires can be found in (www.epi.minsal.cl/encuesta-ens/). Table 1 provides a description of the different modules from which the data were obtained for the development of the study.

**Table 1:** Description of the modules used in the study.

	Modules	Description
**Form 1**	I Characterization of the interviewee	Nationality, language, residence in Chile, belonging to indigenous peoples
	IV Physical activity	Time spent participating in different types of physical activity, such as during work, moving from one place to another, or during free time
	XI Nutritional status	Diagnosis of excess weight and what is being done to reduce excess weight
	XII Diet	frequency of consumption of water, fish, dairy, legumes, fruits and vegetables
	XIII Sleep	Number of hours slept during the week and on weekends, use of sleeping pills, and trouble falling asleep
	XIV Smoking status	Tobacco use, age when started smoking, number of cigarettes, and having tried to quit smoking, among others.
	XX Psychosocial	Thoughts and support in different situations and symptoms of stress or anguish
	XXI Socioeconomic level of the household	Marital status, pension system, highest education level achieved, employment status, socioeconomic status and type of dwelling (water, electricity, human waste disposal and heating).
**Form 2**	VII Alcohol consumption	Consumption of alcohol or alcoholic beverages, quantity and frequency of consumption.

**Source:** Own elaboration based on the ENS.

Because the objective is to analyse the main determinants of the prevalence of overweight and obesity in Chile, the dependent variable is body mass index (BMI). Although this variable can be obtained for each individual as a continuous variable, alternative definitions may be more relevant to achieve the proposed objective. If the variable is considered continuous, the parameters of the explanatory variables introduced in the regression are interpreted as marginal propensities that are constant across the entire sample [25]. That is, the extent BMI increases after an increase in the corresponding explanatory variable, regardless of whether said person is of normal weight, overweight or obese, is measured. Therefore, the determinants of BMI are being measured, not those of overweight and obesity. If 3 categories are defined using BMI (normal weight, overweight and obesity), the appropriate model would be a multinomial logistic regression model. The estimated parameters measure the determining factors associated with each category, but the model is not capable of differentiating between BMI values within each category. That is, a person with a BMI of 26 and another with 29 would be treated in an analogous way, and our objective, however, is to see what factors explain why each one has a different BMI. An alternative that avoids the drawbacks of the 2 previous models consists of considering BMI as a censored variable; that is, censoring values below 25 to zero (normal weight) and continuing from said value, which is the approach adopted in this work. In this case, Heckman’s sample selection model is appropriate.

The traditional approach that has been used to study the relationship between overweight and obesity and its different factors is multiple regression models (adapted to the characteristics of the dependent variable). In the study of a complex phenomenon such as this, these models have some drawbacks, such as i) rigid structures of the models; ii) imposed constraints on the stochastic properties of the parameters and their distributions; and iii) difficulty in considering complex relationships and nonlinear interactions between explanatory variables [26, 27]. ML tools can overcome some of the deficiencies found in the regression models when faced with a large number of explanatory variables and the ways in which they interact with the dependent variable. To facilitate the process of including the explanatory variables that most affect the prevalence of overweight and obesity in Chile, in this study, an ML tool, random forest (RF), was chosen. The advantage of this approach is two-fold. First, it does not impose stochastic assumptions on the relationships between the dependent variable and the independent variables. Second, it allows the possible presence of nonlinear relationships between the dependent variable and the explanatory variables [26, 28]. However, RF has 2 disadvantages: i) it may not efficiently incorporate known causal relationships between the variables because they are based on algorithms that are not supported by underlying theories, and ii) they cannot perform extrapolations through the prediction of data with characteristics outside the range of the samples [28]. For this reason, the combined use of RF and, later, of an econometric model, specifically the Heckman sample selection model, makes it possible to capitalize on the strength of both methods and overcome the corresponding drawbacks [26, 29].

Therefore, in this work, the methodological approach adopted consists of carrying out 2 complementary analyses. In the first stage, RF was used to preselect the main determinants of the prevalence of obesity in Chile as well as the possible interactions between these factors. The objective of this first stage is to facilitate the specifications of the econometric model.

The application of the RF model allows for two main approaches. The first approach, which is the most commonly employed in the literature, involves using RF for prediction when dealing with a large dataset containing a significant number of variables. This approach leverages the inherent strength of RF in handling high-dimensional data and capturing complex relationships. Its ability to manage large datasets with numerous predictors makes it a go-to method for researchers aiming to achieve accurate and reliable predictions. The second approach focuses on variable importance, used when the study’s objective is to identify the most critical variables within a dataset. RF can determine which variables are most influential by calculating the frequency with which each variable is selected across all individual trees in the ensemble. This approach is particularly valuable for feature selection, allowing researchers to narrow down the list of predictors to those that significantly impact the outcome variable.

RF forest model was employed in this second approach (exploratory) to assess the importance of variables and inform the development of subsequent econometric models, Strobl et al. [30] and Genuer et al. [31] has shown the dependability of RF intrinsic indicators of feature importance, which eliminate the requirement for a train-test split when the aim is solely to prioritize predictors. Our primary objective was not predictive accuracy but rather identifying significant predictors for inclusion in our econometric framework. Given the exploratory nature of our analysis and the emphasis on variable selection rather than prediction, we opted not to employ a train-test split. Instead, we utilized the entire dataset for estimation to maximize the information available for model calibration.

**Fig 1:**
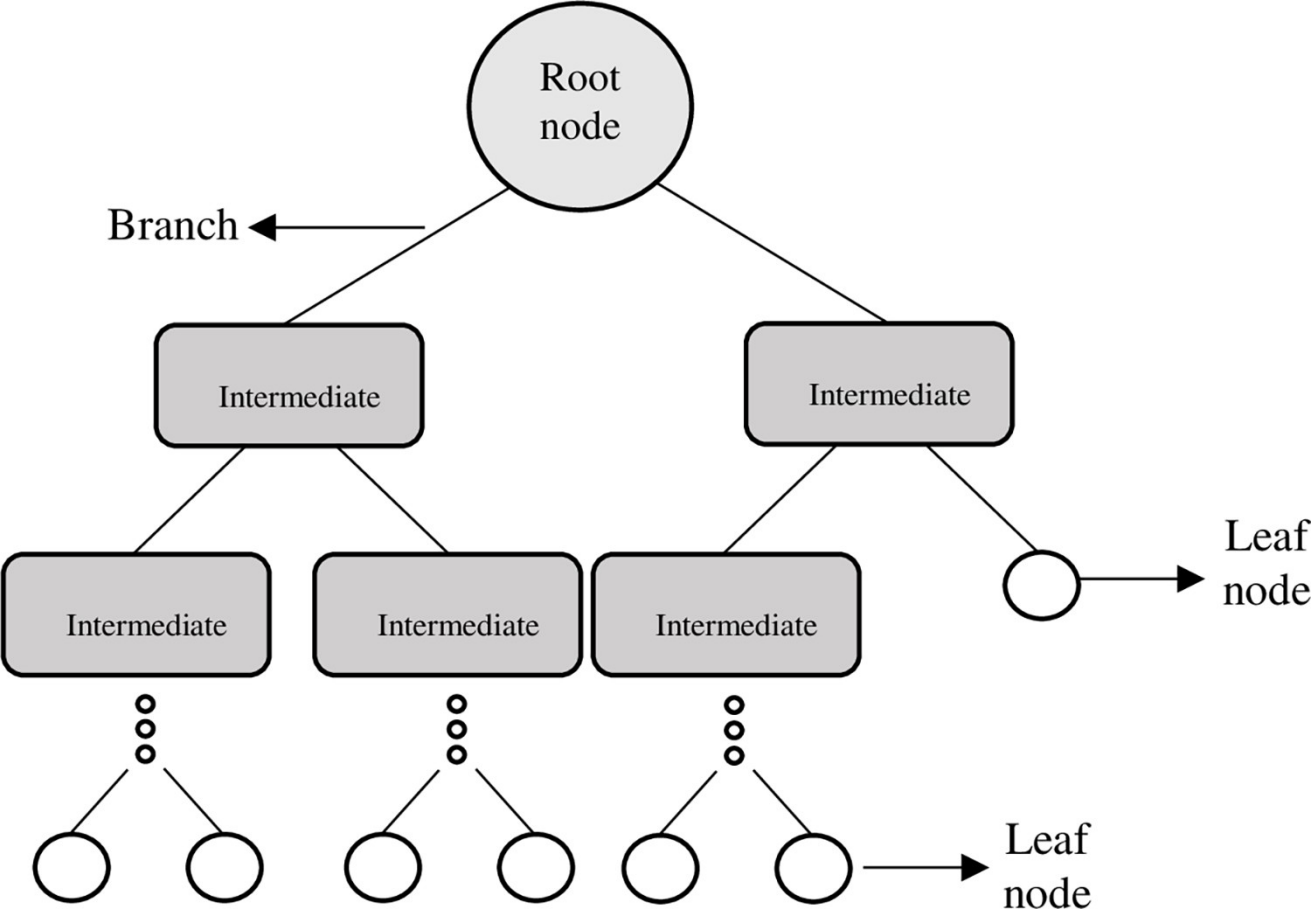
Decision tree diagram. **Source:** Own elaboration.

RF is a supervised learning algorithm developed from a set of multiple decision trees. Decision trees are also a supervised and nonparametric learning algorithm; they are presented as a tree structure (Fig 1) and are mainly composed of 3 elements.

The decision node indicates that a decision must be made; the higher decision node is generally called the root node, and the subsequent nodes are called intermediate nodes. The branches show the different options after making a decision. The leaf or probability nodes are at the lower end of the structure; they represent the results, that is, at that point, a random event occurs with an associated probability. The objective of this algorithm is to classify individuals based on the information available for them for different variables. To achieve optimal precision with the RF model, we used cross-validation with kfold = 5.

For the generation of a decision tree, the Hunt algorithm is used [32]. If all the records of the set belong to the same class, then the node, defined as *t*, is a leaf node; otherwise, if the records are distributed into more than one class, a variable must be chosen to divide the data into smaller subsets. This procedure is applied recursively for each subset of the data. The algorithm is carried out in such a way that the division performed in each stage is optimal according to some impurity criterion. The impurity criteria are entropy, the Gini index and the classification error; however, the latter was not used in this research.

Entropy measures how well an attribute separates observations according to the desired classification and is expressed as [33]:

$$Entropy(t)=-\sum_{i=1}^{c}p(i)\log_{2}p(i)$$ (1)

where *t* represents the subset on which the entropy is calculated, *c* represents the classes of the variable of interest, and *p(i)* is the proportion of observations that belong to class *i* of the response variable being at node *t*.

The Gini index measures the degree of purity of a decision node and is expressed according to the following expression [33]:

$$Gini(t)=1-\sum_{i=1}^{c}{\left(p(i)\right)}^{2}$$ (2)

where the elements *t*, *c*, and *p(i)* represent the same as those in the previous equation.

The algorithm starts with a root node, which is where the first division occurs based on the levels of that variable that minimizes the added impurity on all the nodes that are generated from the division, where this aggregation is constructed as a weighted average of the chosen impurity criterion, weighted by the proportion of records in each node; that is, the variable that minimizes the following expression is selected:

$$\sum_{i=1}^{k}\frac{n_{i}}{n}I(i)$$ (3)

where *I* is the impurity criterion, *i* is each node that was generated from the division, *n*_*i*_ is the number of observations at node *i*, and *n* is the total number of observations.

As an example, suppose we have a dataset corresponding to survey responses, and we want to classify the nutritional status of each individual (underweight, normal, overweight, or obese). In this case, there must be 2 vectors of variables: the first that collects the information of the independent variables for each individual and the second that contains the response variable that corresponds to the classification of the nutritional status for each individual. First, the set of independent variables is entered into the algorithm, and the added impurity is measured on the divisions generated from the levels for each variable. The variable with the lowest added impurity will be the variable chosen to divide the set, and the first division occurs. For example, if the chosen variable is marital status, there would be 2 branches: one would contain married people or couples, and the other branch would contain single people. Once the division of the root node has been selected, the next step is to recalculate the impurity for all the remaining variables in each of the branches, and the variable with the lowest added impurity is chosen again to generate new divisions. Continuing with the example, suppose that for married individuals or couples, the variable that generates the least added impurity is gender; this variable would be part of the next intermediate decision node, where one branch will be for females and the other branch for males. For the other branch of the root node, which corresponds to single persons, the same procedure is carried out, but the variable with the lowest added impurity may be different from that for the married or couples branch. This process continues iteratively for the different branches of the intermediate nodes. The algorithm ends when, for each node, there is no division that allows reducing the added impurity, that is, when it is no longer possible to improve the ability to discriminate individuals on the basis of the response variable. At this point, all the instances of branch choice belong to the same classification category (underweight, normal, overweight or obese) [34]. This result of the classification is represented by the leaf nodes.

The precision of a decision tree is obtained from the percentage of individuals correctly classified according to the categories into which the response variable has been divided. For greater precision, multiple decision trees are combined to form a RF. Each decision tree of the RF uses a random subsample with replacement of the total observations; that is, a tree will depend on the values of a random vector of the sample independently [35], and therefore, no tree will be the same as another within the same RF because they are built with the same variables but different subsamples.

RF classifiers reveal the importance of the variables, which indicates the magnitude of the contribution of each variable to the prediction of the classes [36]. In each decision tree, the variables are scored according to the impurity measure of the decision node. The importance of a variable within the RF is measured as the average of the scores obtained in each tree, and then, it is normalized to 1 [37].

The importance of the variables within the RF is the tool that will help in the construction of the Heckman selection model. For the use of RF, 3 parameters were specified: impurity measure, number of decision trees to be used and maximum depth of the decision tree, that is, the maximum number of vertical levels that the tree can have. The objective is to classify nutritional status (0: underweight or normal; 1: overweight or obese) from one vector of variables that are listed in Table 2.

**Table 2:** Characterization of the sample based on body mass index (BMI).

Factor	Variable (n)	Variable category	Percentage of the sample	BMI mean	p-value
**Sociodemographic**	Gender	Male	36.08	28.32	0.000
	(5 269)	Female	63.92	29.55	
	Marital status	Married or with a partner	49.00	29.67	0.000
	(5 269)	Single	51.00	28.57	
	Age	18–34 years	24.05	27.84	0.000
	(5 269)	35–50 years	24.69	29.71	
		51–64 years	25.85	29.97	
		65 or more years	25.41	28.84	
	Education level	Up to basic education	32.55	30.17	0.000
	(5 223)	Up to middle school	34.94	28.95	
		Professional or postgraduate	32.51	28.20	
	Geographic zone	Northern	26.02	28.73	0.000
	(5 269)	North central	22.09	28.76	
		Metropolitan region	14.97	28.56	
		Southern	26.36	29.87	
		Southernmost	10.55	29.62	
	Geographic area	Rural	16.11	29.94	0.000
	(5 269)	Urban	83.89	28.95	
	Total income	Less than $384,000	57.43	29.59	0.000
	(4 491)	More than $384,000	42.57	28.69	
	Employment situation	Working	47.76	28.93	0.019
	(5 251)	Retired	20.55	29.04	
		Other	31.69	29.42	
	Housing type	Precarious housing	0.51	28.38	0.001
	(5 269)	Semidetached house	6.40	28.90	
		Apartment	47.07	28.42	
		Independent house	46.02	29.42	
	Health system	Public (FONASA)	84.63	29.27	0.000
	(5 213)	Armed Forces-ISAPRE-Other-None	15.37	28.30	
**Behavioural**	Alcohol consumption	Never	35.22	29.44	0.000
	(5 269)	Once a month	36.42	29.48	
		2 to 4 times a month	20.61	28.41	
		2 or more times a week	7.74	27.66	
	Smoking status	Does not smoke	46.80	29.19	0.000
	(5 269)	Quit smoking	24.41	29.56	
		Smokes	28.79	28.60	
	Daily water consumption	No glass	7.86	28.65	0.005
	(5 264)	1–4 glasses	57.18	28.97	
		5–8 glasses	28.02	29.37	
		More than 8 glasses	6.93	29.73	
	Diet	Bad	45.72	29.23	0.139
	(5 269)	Good	54.28	29.00	
	Physical activity	No	79.22	29.45	0.000
	(5 269)	Sometimes	10.61	27.96	
		Yes	10.17	27.63	
	Hours of daily sleep	1–5 hours	13.51	29.70	0.006
	(5 269)	6–8 hours	73.9	29.04	
		More than 8 hours	13.40	28.87	
	Review nutritional label	Never	47.98	29.26	0.126
	(5 269)	Sometimes	21.22	29.03	
		Always	30.80	28.92	
	Review warnings	Never	41.01	29.14	0.562
	(5 269)	Sometimes	18.49	29.23	
		Always	40.50	29.01	
**Psychosocial**	Stress	Never	39.37	29.15	0.030
	(5 268)	Sometimes	39.27	29.13	
		Several times	15.64	28.70	
		Always	5.71	29.77	
	Financial stress	Little or nothing	45.82	28.86	0.001
	(5 212)	Moderate or high	54.18	29.36	
	Emotional support (5 241)	No	14.75	29.36	0.179
		Yes, sometimes	22.74	29.25	
		Yes, always	62.51	29.01	

**Source:** Own elaboration.

Three RF analyses were performed, each consisting of a vector of independent variables different from each other. To achieve the best RF, in terms of precision, for each vector of variables, different tests were run using RFs with 50, 100, 200, 400, 600, 800, 1000 and 1200 decision trees; Gini and entropy impurity criteria; and a maximum depth of 20 levels. In each analysis, the RF with greatest precision was selected, that is, the one that performed best with the previously incorporated variables.

In the first analysis, each of the variables in Table 2 were used without considering interactions, obtaining a precision of 77.24%. The variables total income, financial stress, gender, marital status, geographic area and pension system had the least explanatory power in terms of classifying nutritional status. In the second analysis, interactions between those variables that had little relevance were considered, for example, gender and marital status as well as financial stress and total income. In this second model, individual variables and interactions were introduced. The joint precision obtained was 72.17%, and the variables included individually were found to have a lower importance index compared to those for the aforementioned interactions. In a third analysis, only the aforementioned interactions were included, and the individual effects corresponding to the variables gender, marital status, financial stress and total income were excluded. For this third analysis, the precision obtained was 77.38%, and the interaction of the variables had a higher importance index. In practical terms, while Random Forests can capture interactions within the data implicitly, the explicit extraction and examination of relevant interactions often require additional steps. These can include creating interaction terms manually and examining their importance or employing techniques to identify and visualize interactions that are particularly influential. Therefore, in our second analysis, we aimed to highlight these specific interactions to provide a more detailed understanding of the relationships between variables, supplementing the inherent capabilities of the RF algorithm.

In 2022, Roman Hornung introduced a new method for explicitly capturing interactions in random forests through the "Diversity Forest" package in R [38]. This approach offers a promising avenue for enhancing the modeling of interactions within random forests. However, this method was not utilized in the analysis presented in this research. Instead, traditional RF techniques was applied and complemented them with an additional focus on specific interactions to better understand their influence.

Once the main determinants of the prevalence of overweight and obesity in Chile are sorted on the basis of their measure of impurity, in a second stage, we measure the magnitude of the effect through the estimation of an econometric model. Taking into account the censored nature of the dependent variable, the Heckman sample selection model was used [39]. This model breaks down the analysis into 2 parts. First, a selection equation is specified that seeks to identify the factors determining whether a person is overweight or obese, regardless of their BMI, compared to people who are normal or underweight. Therefore, it is a logistic regression in which the dependent variable takes the value 1 if the person is overweight or obese and zero otherwise. In the second equation, conditioned by the first, for those who are overweight or obese, main determinants of the BMI (defined as a continuous variable) are considered. The explanatory variables in the 2 stages may be different. The first equation, or selection equation, is defined as follows:

$$z_{i}^{*}=w\prime \gamma_{i}+u_{i}$$ (4)

where

$z_{i}^{*}$ is a latent variable that represents the nutritional status for individual *I*;

*w*′ is the estimate of the coefficients of the selection equation;

*γ*_*i*_ it is a vector of characteristics of the individual; and

*u*_*i*_ is an error term with a normal distribution.

The second equation, or regression equation, is defined as follows:

$$y_{i}=x_{i}^{\prime }\beta +\varepsilon_{i}$$ (5)

where

*y*_*i*_ corresponds to the BMI for individual *i* of people with overweight and obesity;

*x*_*i*_ it is a vector of characteristics of the individual;

*β* is the estimation of the coefficients of the regression equation; and

*ε*_*i*_ is an error term with a normal distribution.

When combining the 2 equations, it is necessary to take into account that the sample is not the same in the 2 choices; therefore, the second equation is given by [40]:

$$E[y_{i}|y_{i}\;\mathrm{is}\;\mathrm{observed}]=x_{i}^{\prime }\beta +\beta_{\lambda }\lambda_{i}(\alpha_{u})$$ (6)

where

$$\alpha_{u}=-\frac{w_{i}^{\prime }\gamma }{\gamma_{u}}$$ (7)

$$\lambda_{i}(\alpha_{u})=-\frac{\varphi (w_{i}^{\prime }\gamma /\sigma_{u})}{\Phi (w_{i}^{\prime }\gamma /\sigma_{u})}$$ (8)

The expression *λ*_*i*_(*α*_*u*_) corresponds to the inverse Mills relationship, which is the probability that an individual is overweight or obese, over the cumulative probability of the decision of an individual in the sample. Therefore, Eq [6] shows that BMI can only be observed when the nutritional status is positive, $z_{i}^{*}>0$. If sample selection bias is omitted, inconsistent and biased estimators are generated [39].

The previous model can be estimated by maximum likelihood or by the two-stage method [39, 41]. In this research, maximum likelihood estimation was chosen because in the absence of multicollinearity, the estimator is more efficient than that using the two-stage method [42, 43] (IBM SPSS Statistics (version 25); Jupyter Notebook (version 6.3.0); Gretl (version 1.9.4) and R Studio (version 4.2.0) software were used).

## 4 Results

The results of this study are presented in 3 parts. First, some descriptive results of the variables included in this work are shown to contribute to a better understanding of the results obtained in the analytical part. In the second part, the relative importance of the different explanatory variables is analysed when classifying the nutritional status in Chile based on RF. Finally, the results obtained from the estimation of the Heckman sample selection model are presented.

Table 2 shows the characterization of the sample based on BMI. Sociodemographic variables, behavioural factor variables and psychosocial factor variables were included. For each variable, an analysis of variance (ANOVA) was performed in relation to BMI to detect significant relationships. As seen, in all cases, except for diet, reviewing the nutritional label, reviewing the warnings and emotional support, significant differences were found between the categories that define each explanatory variable. The average BMI of the sample was 29.11, which corresponds to a nutritional status of overweight.

In the sample, women had a higher average BMI than did men. Regarding marital status, people who were married or in a relationship had an average BMI of 29.67, above the average value for those who were single. Regarding age range, people between 51 and 64 years had a higher average BMI. Additionally, social class was an important determinant of the population’s BMI. Individuals with a lower level of education had an average BMI of 30.17, which corresponds to obesity. The relationship between higher body mass indices among individuals in lower social classes has been reported by other studies that focused on different developed countries belonging to the OECD [44].People residing in the southern part of the country had a higher average BMI, while physically inactive people had an average BMI of 29.45, a figure that exceeded that of people who performed physical activity by 2 points.

Once the sample was characterized and the causal relationships between the different explanatory variables and BMI were analysed, RF analysis was applied to prioritize the different explanatory variables according to the impurity criterion. Table 3 shows the final RF results.

**Table 3:** Results of the application of the random forest (RF) method to determine the relative importance of the different variables and their interactions in the explanation of the nutritional status in Chile.

Ranking of variables	
(Importance index)	
1	Geographic zone (0.0828)
2	Financial stress x Total income (0.0723)
3	Stress (0.0701)
4	Alcohol consumption (0.0683)
5	Daily water consumption (0.0647)
6	Gender x Marital status (0.0640)
7	Age (0.0625)
8	Smoking status (0.0590)
9	Emotional support (0.0545)
10	Nutritional label review (0.0486)
11	Warnings review (0.0482)
12	Type of dwelling (0.0472)
13	Daily hours slept (0.0466)
14	Education level (0.0461)
15	Work situation (0.0450)
16	Diet (0.0427)
17	Physical activity (0.0320)
18	Geographic area (0.0237)
19	Pension system (0.0219)

**Source:** Own elaboration.

Accuracy estimate: 77.38%

Standard Deviation: 0.0052

Impurity criterion: Entropy

Number of trees: 400

Finally, with these results, a censored regression model was specified and estimated by applying the Heckman sample selection method to quantify the impact of each variable on the BMI in Chile. As mentioned in the previous section, estimations were performed in 2 stages. In the first stage, selection model estimation was carried out exclusively using the sociodemographic variables, and in the second stage, the specification included the entire set of variables. In this case, a stepwise process was followed; that is, the model was first estimated by introducing the variable with the greatest relative importance as determined from the RF. Next, the second variable was introduced to see the change in the fit indicators and the increase in explanatory power. If the explanatory power did not increase, the variable was excluded, and the next variable was introduced. Once all the significant variables were incorporated, the least significant ones were excluded until a set of variables was reached that maximized the explanatory power of the model. Table 4 shows the results of the selection model estimation (first equation).

**Table 4:** Results of the Heckman selection model estimation.

Variable	Variable category	Beta (Standard error)
Constant		-0.06 (0.33)
Gender x Marital status	Married man or couple	0.30*** (0.07)
	Married woman or couple	0.45*** (0.07)
	Single woman	0.19*** (0.06)
Age	18–34 years	-0.15* (0.08)
	35–50 years	0.16** (0.08)
	51–64 years	0.19** (0.08)
Educational level	Up to basic education	0.27*** (0.06)
	Up to secondary or technical education	0.08 (0.05)
Geographic zone	Northern	-0.15** (0.06)
	Downtown area	-0.25*** (0.06)
	Metropolitan region	-0.06 (0.07)
	Southernmost	-0.14* (0.08)
Geographic area	Urban	-0.05 (0.07)
Employment situation	Working	0.14*** (0.05)
	Retired	0.03 (0.08)
Type of dwelling	Independent house	0.48 (0.3)
	Semidetached house	0.54* (0.3)
	Apartment	0.45 (0.31)

**Source:** Own elaboration

Dependent variable: underweight-normal weight (0), overweight-obese (1)

Sample size = 4 367

* p<0.1

** p<0.05

*** p<0.01

As seen in the selection model, almost all of the variables were found to be significant. Because it is a probabilistic model, the coefficients indicate the probability that a person with a certain characteristic is overweight or obese relative to a person is normal or low weight. All the explanatory variables are categorical; therefore, the marginal effects have been calculated, which can be interpreted in a similar way to the parameters estimated in a regression. Table 5 provides the results.

**Table 5:** Average marginal effects obtained from the parameters in Table 4.

Variable	Variable category	Marginal effect (dy/dx) (Standard error)
Gender x Marital status	Married man or couple	0.08*** (0.02)
	Married woman or couple	0.12*** (0.02)
	Single woman	0.05*** (0.02)
	Single man^**Rc**^	
Age	18 to 34 years	-0.04 (0.03)
	35 to 50 years	0.05** (0.02)
	51 to 64 years	0.05*** (0.02)
	65 or more years^**Rc**^	
Education level	Up to basic education	0.07*** (0.02)
	Up to secondary or technical education	0.02 (0.01)
	Professional or postgraduate^**Rc**^	
Geographic zone	Northern	-0.05** (0.02)
	Downtown area	-0.08*** (0.02)
	Metropolitan region	-0.02 (0.02)
	Southern	-0.04* (0.02)
	Southernmost^**Rc**^	
Geographic area	Urban	-0.01 (0.02)
	Rural^**Rc**^	
Employment situation	Working	0.04*** (0.02)
	Retired	0.01 (0.02)
	Other^**Rc**^	
Type of dwelling	Independent house	0.13* (0.08)
	Semidetached house	0.15* (0.08)
	Apartment	0.10* (0.06)
	Precarious housing^**Rc**^	

**Source:** Own elaboration.

Sample size = 4 367

* p<0.1

** p<0.05

*** p<0.01

**Rc** = Reference category

The results indicate that married women or those in a relationship had a greater increase in the average probability of being overweight or obese, with an increase of 12%. Married men or partnered men and single women had a higher average probability of being overweight or obese by 8% and 5%, respectively, compared to single men, which is the base category. Regarding age, people between 35 and 50 years had an increased probability of being overweight or obese, by 5%, on average, which increased slightly for people between 51 and 64 years (5%), always in relation to the base category (people aged 65 or over). Regarding education level, people with the lowest educational level had a 7% higher probability of being overweight or obese than did those with the highest level of education. Regarding geographical zone, in almost all areas, except for the metropolitan region, the average probability of being overweight or obese decreased, being greater in the downtown zone, with a decrease of 8% compared to the southern zone. For people who worked, the average probability of being overweight or obese increased by 4% compared to those who in school, retired, or other. Finally, in relation to dwelling type, the results indicated something similar to those obtained for social class. Compared to the base category (precarious housing), people who lived in other types of housing had a greater probability of being overweight or obese, being higher among those who lived in semidetached houses (15%).

Table 6 presents the results of the second equation. First, the inverse relationship of the Mills ratio (Lambda) was negative and significant, indicating that there was a sample selection bias, which indicates that the procedure used to estimate the determinants of the prevalence of overweight and obesity in Chile was correct.

**Table 6:** Results of the estimation of the Heckman regression model.

Factor	Variable	Variable category	Regression variable BMI	Standardized betas
			beta (Standard error)	
**Sociodemographic**	Constant		28.98*** (0.54))	
	Gender x Marital status	Married woman or couple	0.92*** (0.22)	0.09
		Single woman	0.69*** (0.21)	0.07
	Age	18–34 years	1.20*** (0.29)	0.10
		35–50 years	1.40*** (0.27)	0.13
		51–64 years	0.99*** (0.24)	0.09
	Education level	Up to basic education	1.17*** (0.25)	0.12
		Up to secondary or technical education	0.30 (0.21)	0.03
	Geographic zone	Northern	-0.95*** (0.31)	-0.08
		Downtown area	-0.98*** (0.31)	-0.08
		Metropolitan region	-1.02*** (0.33)	-0.08
		Southern	-0.38 (0.30)	-0.04
	Geographic area	Urban	0.01 (0.26)	0.00
	Employment situation	Working	-0.71*** (0.20)	-0.07
	Type of dwelling	Independent house	0.17 (0.19)	0.02
		Apartment	0.35 (0.37)	0.02
	Pension system	FONASA	0.30 (0.23)	0.02
**Behavioural**	Alcohol consumption	2 or more times a week	-0.58** (0.27)	-0.03
	Smoking status	Quit smoking	0.27 (0.18)	0.02
	Daily water consumption	5–8 glasses	0.36** (0.18)	0.03
	Diet	Bad	0.17 (0.17)	0.02
	Physical activity	No	0.71*** (0.20)	0.06
	Hours of daily sleep	1–5 hours	0.44* (0.24)	0.03
	Nutritional label review	Never	0.76** (0.33)	0.08
	Warnings review	Never	-0.66* (0.34)	-0.07
**Psychosocial**	Stress	Permanently	0.62* (0.37)	0.03
	Financial stress x Total income	Moderate or high with income greater than $ 384 000	-0.40* (0.20)	-0.03
	Emotional support	Sometimes	0.35* (0.21)	0.03
	Lambda		-0.28* (0.17)	

**Source:** Own elaboration

Log-Likelihood: -12 289.83

Number of observations: 4 367

Censored observations: 973 (22.3%)

Mean BMI: 31.1131

Sigma: 4.6968

Rho: -0.0599

SBIC: 24 815.41| AIC: 24 637.65 | HQC: 24 701.19

* p<0.1

** p<0.05

*** p<0.01

Regarding the sociodemographic factors, most of the variables related to socioeconomic aspects were significant. Among these variables, the first 3 (gender interacting with marital status, age and education level) need to be addressed because the results obtained in this study differ from those obtained for other countries. Regarding gender and marital status, being a woman and being married or living with a partner positively affected BMI (0.92 points greater than the other categories). The literature is not very conclusive on this point, although the studies analysed do not investigate the gender-marital status interaction but rather focus exclusively on the results by gender. Some studies report that women have a higher prevalence of overweight and obesity than do men, and this fact is more accentuated in obesity [2]. However, in 2 studies of OECD countries [45] and 16 European countries [46], the opposite conclusion was reached, with a higher prevalence of obesity and overweight among men.

Regarding age, there is a consensus in the literature that overweight and obesity increase with age. For Chile, the result obtained is similar, but only up to the age range between 35 and 50 years, in which the difference between the BMI of the older population is 1.40, decreasing for the successive age ranges. Additionally, in this case, the results differ from those of other studies. For example, descriptive results reported in some studies for OECD countries indicate that the prevalence of obesity gradually increases until age 59 (26.17%) and then decreases [47, 48]. In countries with higher incomes, the prevalence increases up to 64 years (with 37.85%) and then decreases [47].

Regarding level of education, there was an inverse relationship between this variable and BMI. Indeed, people with a lower level of education presented, on average, BMI values 1.17 points above those with more education (secondary, technical, professional or postgraduate). These results are similar to those reported in other studies in Chile [49] and worldwide, where a lower education level is related to a higher BMI [44]. In all these studies, a higher level of education is associated with a greater awareness of problems related to health and especially to diet.

For the behavioural variables, the results obtained in this study are similar to those previously reported in the literature both for Chile and for other countries. We highlight in particular the results related to water consumption, physical activity and hours of sleep [3, 14, 50], i.e., the highest estimated parameters. For the last 2 variables, the relationship was negative; that is, less physical activity and fewer hours of sleep were associated with higher body mass indices (0.71 and 0.44 points for those who do some type of physical exercise or sleep more hours, respectively). The relationship, in contrast, with water consumption was positive; that is, with increased consumption (between 5 and 8 glasses per day), the BMI increased 0.36 points. Regarding alcohol and cigarette consumption, some differences were observed in relation to what was reported in the literature. For alcohol consumption, an inverse relationship with BMI was observed, a finding that is consistent with most results reported in the literature; however, we also found evidence to the contrary. Similar to the results obtained in other studies, worldwide evidence is not conclusive with regard to this relationship [51]. For smoking, although those who have stopped smoking presented an increase in BMI of 0.27 points, this difference was not significant. This may be because the downwards trend in tobacco consumption has been significant and continuous since 2009; therefore, it does not currently represent a differential behaviour [10]. Finally, for the 3 behavioural variables related to diet, the results showed the expected signs. Of particular interest is the attention given to information on labels. Those people who do not pay attention to the warnings included on packages (high in saturated fat, high in sugar, high in sodium, high in calories, etc.) had a lower BMI, by 0.66 points. Those consumers who do not pay attention to the nutritional content on labels had a higher BMI, by 0.76 points. These 2 results are particularly interesting because they allow us to reflect on the impact that the regulation imposed by the government of Chile has had on reducing the prevalence of overweight and obesity (Law 20 606 of 2012). Unlike other studies [3, 14, 50], the quality of the diet did not have a significant effect on the BMI of the Chilean population. The effect was as expected; that is, the worse the quality of the diet, the higher was the BMI, but the parameter (0.17) was not significant.

All the variables included within the psychosocial factor were significant. Regarding stress and emotional support, a positive relationship was observed with the BMI of the Chilean population. These variables have rarely been considered in the previous literature; however, as in this study, they are clearly determining factors of the level of overweight and obesity in the population, making it necessary to incorporate them in future studies, as has already been done in recent studies [18–20]. Finally, we would like to highlight the result obtained for the interaction between financial stress and total income. This interaction has rarely been considered in the literature; however, it was the second most important variable in explaining the prevalence of overweight and obesity, as determined in the RF analysis. The results obtained indicate that those people who suffer moderate or high financial stress but who have a higher income had a lower BMI. This result is very interesting because the results in Table 2 indicated that the average BMI of the people who presented financial stress was higher than that of the rest. However, when interacting with income, this relationship was only positive if the person who presented this stress had a low income level, a result similar to that found in Garasky et al [21].

## 5 Concluding remarks

This paper aimed at analysing the key determinants for the prevalence of overweight and obesity in Chile. In addition to traditional factors that were considered in the previous literature (such as sociodemographic and behavioural factors), in this study we have tested the potential relevance of new variables that have been recently considered such as stress, financial stress and emotional support. Moreover, we have considered the potential interactions among the set of variables used in this study.

The main methodological contribution of this paper relies on the use of a mixed strategy combining artificial intelligence and econometric tools. Among the former, we have chosen the random forest tool in order to make a pre-selection of potential variables which have an impact on the prevalence of overweight and obesity in Chile. The chosen variables were then considered in the econometric model. As we were interested only in overweight and obese population, a sample selection econometric model has been used, which is a second methodological contribution of this paper.

Results obtained suggested a number of issues. First, our results are consistent with previous literature on the effects of traditional variables such as, age, education level, water consumption, physical activity and hours of sleep on the prevalence of overweight and obesity [17–19], and also with previous research in Chile [16]. However, this study has considered a large range of potential variables as well as their interaction, which have allowed to show new interesting results. Particularly, we have highlighted some significant effects of factor like gender in interaction with marital status, stress, financial stress in interaction with income or emotional support. These variables should be also considered in further research when designing longitudinal analyses. Finally, there is an interesting result for policy analysis. Despite the fact that the Chilean food labelling law is one of the most demanding public policies in the world, our results indicate that paying attention to the nutritional and warning claims does not have a significant effect on reducing the prevalence of overweight and obesity. Although taking into account the dataset we have considered in this study, it not possible to evaluate the effectiveness of the Chilean labelling policy, this result, at least, suggests that further research is needed on how consumers perceive such information and to what extent the contribute to behavioural change.

## List of abbreviations

ENS: National Health Survey
RF: Random Forest
WHO: World Health Organization
MINSAL: Ministry of Health
OECD: Organization for Economic Co-operation and Developmen
ML: Machine Learning
ANOVA: Analisys of Variance
